# Glowstick-inspired smartphone-readable reporters for sensitive, multiplexed lateral flow immunoassays

**DOI:** 10.1038/s44172-023-00075-2

**Published:** 2023-06-23

**Authors:** Kristen Brosamer, Katerina Kourentzi, Richard C. Willson, Binh V. Vu

**Affiliations:** 1 Department of Biomedical Engineering, University of Houston, Houston, TX 77204, USA.; 2 William A. Brookshire Department of Chemical and Biomolecular Engineering, University of Houston, Houston, TX 77204, USA.; 3 Escuela de Medicina y Ciencias de Salud, Tecnológico de Monterrey, Monterrey, Nuevo León 64710, Mexico.

## Abstract

The COVID-19 pandemic has increased demand for point-of-care (POC) screening tests such as lateral flow assays (LFAs) and highlighted the need for sensitive and cost-effective POC diagnostic platforms. Here, we demonstrate an LFA platform using standard fluorescent nanoparticle reporters in which optical excitation is replaced by chemical excitation using the peroxyoxalate-based chemistry of inexpensive, shelf-stable glowsticks. The one-step chemi-excitation of fluorescent particles produces visible light readable by an unmodified smartphone, enhancing sensitivity while preserving simplicity and cost-effectiveness. Our Glow LFA detected the common model analyte human chorionic gonadotropin with a limit of detection (LoD) of 39 pg/mL—over ten times more sensitive than standard gold nanoparticles using the same antibodies. We also demonstrate its application to the detection of SARS-CoV-2 nucleoprotein at 100 pg/mL in nasal swab extract. Multiple fluorescent dyes can be chemi-excited by a single reagent, allowing for color multiplexing on a single LFA strip with a smartphone camera. The detection of three analytes on a single LFA test line was demonstrated using red, green, and blue fluorescent reporter particles, making glow LFA a promising platform for multiplexed detection.

The COVID-19 pandemic has emphasized the need for rapid, inexpensive, and ultrasensitive immunoassays for point-of-care (POC) diagnostic applications^1^. Lateral flow immunoassays (LFAs) such as the home pregnancy test and COVID-19 rapid antigen test are successfully used by untrained persons to detect medically-important analytes, but have limited analytical sensitivity and typically detect only a single analyte^2,3^.

Even with optimized antibodies and buffers, the analytical sensitivity of an LFA is largely limited by the detectability of the reporter particles^4^. Typical colorimetric gold or latex LFA reporters are insufficiently detectable for many clinical applications, such as diagnosing the early stages of viral infection^4^. Fluorescent reporters, including organic fluorophores^4–8^, europium chelates^9,10^, up-converting phosphors^11,12^, and quantum dots^13^ provide increased analytical sensitivity but require complex detection optics. Many fluorescent reporters require multiple sets of narrow-bandpass filters and do not lend themselves to a multiplex format^14^. Signal amplification by enzymes or enzyme-mimics (e.g., horseradish peroxidase^15^, Pt nanoparticles^16,17^) or by analyte (e.g., nucleic acid) amplification^18^ can increase LFA sensitivity but these formats cannot readily be multiplexed, require complex workflows, and use reporters or enzyme substrates that often are not stable at room temperature^19^, thereby limiting their POC utility. Additionally, multiplex POC assays remain challenging to develop^20,21^. High-sensitivity, optically-excited LFA reporters (e.g., fluorophores) require specific excitation/emission optics, increasing cost and complexity and impairing multiplex application. Thus, despite multiple advances, there is still a need for more-sensitive, multiplexable LFA technologies with practical ease of use, shelf-stability, and reading simplicity.

Building on the stable, inexpensive peroxyoxalate chemistry of common glowsticks^22^, we here introduce chemi-excited “glow” LFA reporters—fluorescent nanoparticles chemically-excited to emit light—that can be readily imaged using a smartphone camera. These reporters provide increased sensitivity and color multiplexing using only a smartphone, a simple plastic dark box, and inexpensive reagents with excellent shelf stability. Glowsticks contain an inner glass ampoule that when broken releases a diphenyl oxalate (e.g., bis[2,4,6-trichlorophenyl] oxalate; TCPO) dissolved in an organic solvent. The oxalate reacts with hydrogen peroxide in a cosolvent surrounding the glass ampoule to produce unstable 1,2-dioxetanedione, a strained cyclic dimer of CO_2_ (Fig. 1a). Cleavage of dioxetanedione to CO_2_ chemically excites soluble fluorophores in the cosolvent, leading to light emission^23,24^. A broad range of organic fluorophores can be excited by the same activating chemicals, and chemi-excitation of fluorophores provides detection sensitivity comparable to complex optical excitation^25^.

Here, we demonstrate fluorescent reporter-based glow LFAs (Fig. 1 and Supplementary Fig. 1) that do not require optical excitation, using chemi-excitation reagents optimized for low toxicity and odor and compatible with nitrocellulose membranes and common clinically-relevant sample matrices (Fig. 1b^26^). In proof-of-concept experiments, we detected human chorionic gonadotropin (hCG) in buffer and SARS-CoV-2 nucleoprotein in nasal swab extract at 39 pg/mL and 100 pg/mL, respectively. We also demonstrate color multiplex detection of multiple analytes on a single LFA test line using a single excitation reagent and an unmodified smartphone.

## Results

### Chemi-excitation of fluorescent particles.

**Fig. 2:**
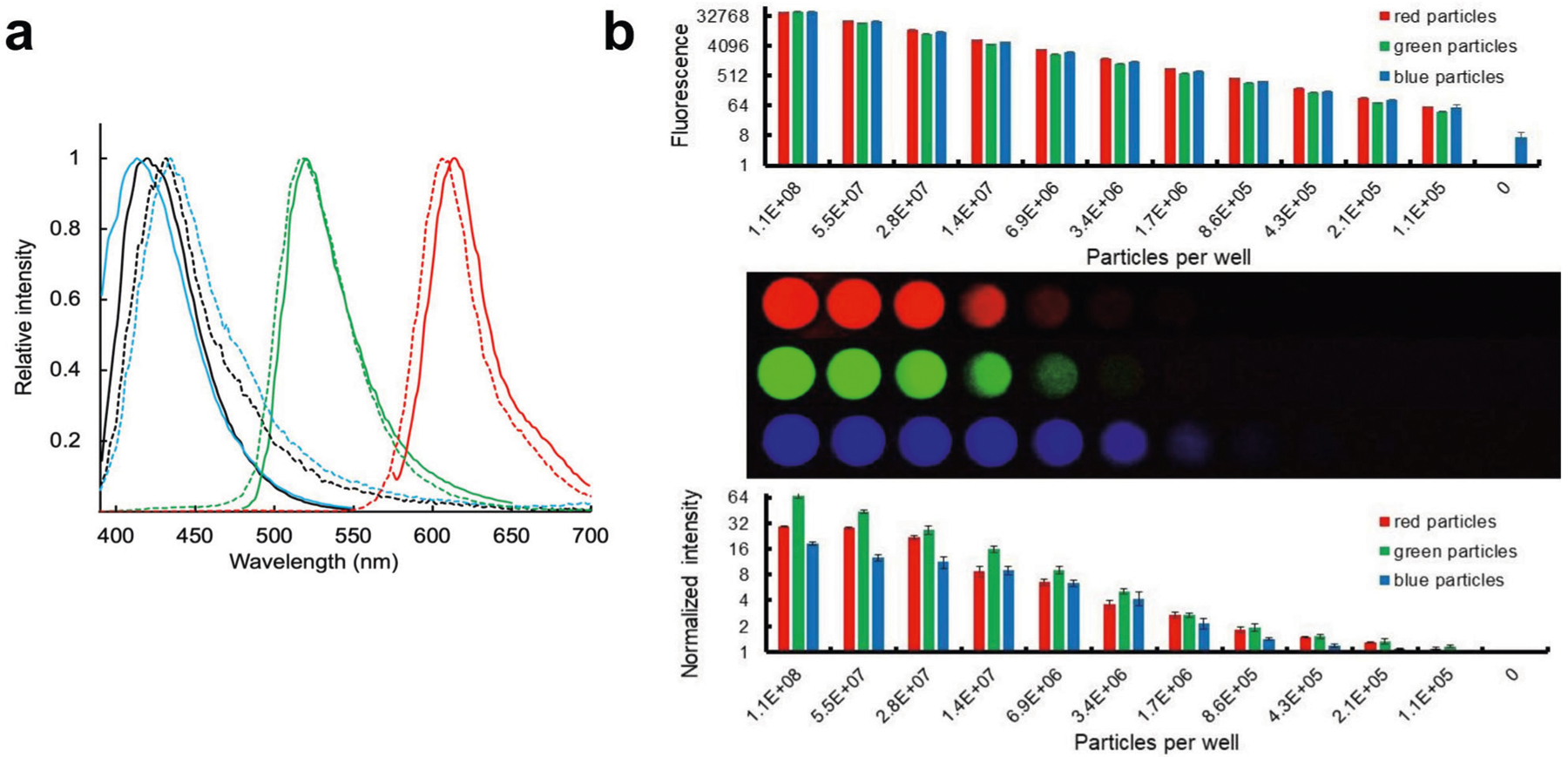
Glow chemi-excitation of fluorescent particles. **a** Emission spectra of optically-excited (dashed lines) and chemi-excited (solid lines) 0.2 μm FluoSpheres Microspheres (blue, Excitation 365 nm/Emission 415 nm; green, Excitation 505 nm/Emission 515 nm; red, Excitation 580 nm/Emission 605 nm; 2 × 10^10^ particles in 50 μL butyl benzoate) and 0.28 μm latex particles in-house dyed with 9,10 diphenylanthracene (Excitation 350–395 nm/Emission 400–450 nm; black lines). Optically-excited emission spectra were obtained with a Tecan M200PRO microplate reader. Chemi-excitation emission spectra were obtained by mixing particles with TCPO/H_2_O_2_ and recording emission spectrum with excitation set to irrelevant 850 nm. **b** Detectability of chemi-excited fluorescent particles by a smartphone camera versus optically-excited fluorescent particles by a Tecan M200PRO plate reader. Top: Optically-excited fluorescence (log scale) of a dilution series read using a plate reader. FluoSpheres Microspheres (blue, green, and red particles as described in (**a**) were twofold serially diluted into a black 96-well plate and measured at their corresponding excitation and emission wavelengths (*n* = 3; average ± 1 Std Dev). Middle: FluoSpheres Microspheres chemi-excited with glow excitation solution and imaged with a smartphone (*n* = 3; average ± 1 Std Dev). Bottom: Intensity profiles (log scale) for chemi-excited particles were extracted from images above (**b**) using ImageJ and normalized by the no-particle signal as described in Methods.

To demonstrate the feasibility of using peroxyoxalate chemistry to chemically excite fluorescent nanoparticles, we swelled and fluorophore-labeled polystyrene particles following the protocol of ref.^27^ (see Methods). The commonly used peroxyoxalate-compatible dye, 9,10-diphenylanthracene (blue, Excitation 350–395 nm/Emission 400–450 nm in ethanol)^28,29^, with a high quantum yield (0.82–0.95 depending on solvent)^30^ and a narrow spectral emission, was chosen as a proof-of-concept for glow reporter particles in LFAs. Peroxyoxalate-based chemi-excitation of 9,10-diphenylanthracene is highly efficient, and we initially demonstrated that the dye alone can be detected down to 200 nM when excited with our in-house glow excitation solution and imaged with a smartphone camera (Supplementary Figs. 2 and 3). Chemi- excited 280-nm polystyrene particles dyed in-house with 9,10-diphenylanthracene also emitted light readily detectable in a microplate reader, with an emission spectrum closely matching the optically-excited emission spectrum of the dye (Fig. 2a).

We found that commercially-available 0.2 μm fluorescent polystyrene ThermoFisher FluoSpheres^™^ Microspheres with different colors (blue: Excitation 365 nm/Emission 415 nm, green: Excitation 505 nm/Emission 515 nm, and red: Excitation 580 nm/Emission 605 nm) also were chemi-excited by a 1:1 mixture of 15 mM TCPO and 3% H_2_O_2_. Upon chemi-excitation, the particles emitted visible light closely matching their emission spectra when optically excited (Fig. 2a).

To assess detection by smartphone camera, FluoSpheres with varying emission spectra were serially diluted into white microwell plate wells, activated with glow excitation solution, and imaged with an unmodified Samsung Note 8 smartphone camera using a simple 3D-printed dark box (coordinates available at https://www.thingiverse.com/thing:5178342/files^31^) (Fig. 2b—Middle and Supplementary Fig. 2). For all three Fluospheres (Red, Green, Blue), we detected ~100,000 particles with chemi-excitation and smartphone imaging (Fig. 2b—Middle/Bottom), sensitivity comparable to that of light-excited readout using a Tecan M200 plate reader (Fig. 2b- Top). This compares favorably with previously reported reader-enhanced detection sensitivities of other LFA reporters, such as thermal contrast amplification detection of gold nanoparticles^32^ (10^5^–10^6^ particles) and magnetic particles^33^ (~10^6^ particles detected by a portable magnetometer).

We typically image the strips within 60 s of the addition of the glow excitation solution. The duration of the glow signal was assessed by chemi-exciting particles in the wells of a 96-well plate and repeatedly imaging with a smartphone over 10 min (Supplementary Fig. 4). The brightness of the glow signal using our current formulation is stable for at least 10 min for red and green particles and declines by 50–60% over 5 min for the blue particles, long enough for convenient reading while maximizing brightness.

### Optimization of the glow chemi-excitation reagent.

A glow-stick’s inner glass tube contains hydrophobic fluorophores and an oxalate such as TCPO dissolved in a nonpolar solvent. The solvent is often diethyl phthalate, which has a low exposure threshold limit value of 0.55 ppm and has been associated with long-term reproductive toxicity^34^. Commercial glowsticks are manufactured from solvent-resistant materials and are fully enclosed to shield users from toxicity and odor. In fully-realized glow LFAs, reagent blisters and a closed cassette will enclose the solvents, minimize user exposure, and improve POC usability^35^. For the near term and for optimal safety and usability, however, solvent toxicity and odor should be minimized. We screened TCPO solvents and H_2_O_2_ cosolvents to minimize toxicity and odor and maximize glow signal strength and stability. Toxicity was evaluated based on US Occupational Safety and Health Administration occupational exposure limits and odor screening used information from The Good Scents Company Information System^36,37^. Signal strength was determined by spotting fluorescent particles on a nitrocellulose membrane (Cytiva; FF80HP; #13549206), chemi-exciting the particles with 15 mM TCPO in the varied solvents mixed with 3% H_2_O_2_ in the cosolvents, and imaging as described in Methods; details of the solvent/cosolvent screening are shown in Supplementary Table 1.

The stability of the formulations was determined by the signal strength in weekly LFA-based testing (see Methods). The signal strength is dependent on the TCPO concentration of the excitation solution. However, the TCPO concentration is limited by its solubility in the chosen solvent. The optimized TCPO solution chosen for glow LFA was 15 mM TCPO dissolved in 33.3% butyl benzoate and 66.6% tributyl citrate. The final H_2_O_2_ substrate contained 45 mL of tert-butanol, 5 mL of 30% H_2_O_2_ (3% final), and 1 mM tetrabutylammonium hydrogen sulfate base catalyst. A mixture of tributyl citrate and butyl benzoate was chosen because each has low toxicity and a mild, sweet-smelling odor, and when used together, they resulted in a stable signal and sharp LFA lines with minimal smearing or displacement of particles. Tert-butanol was chosen as H_2_O_2_ cosolvent because it produced a longer-lasting signal (>5 min) than other candidates.

### Application of chemi-excited glow reporters in LFA.

**Fig. 3:**
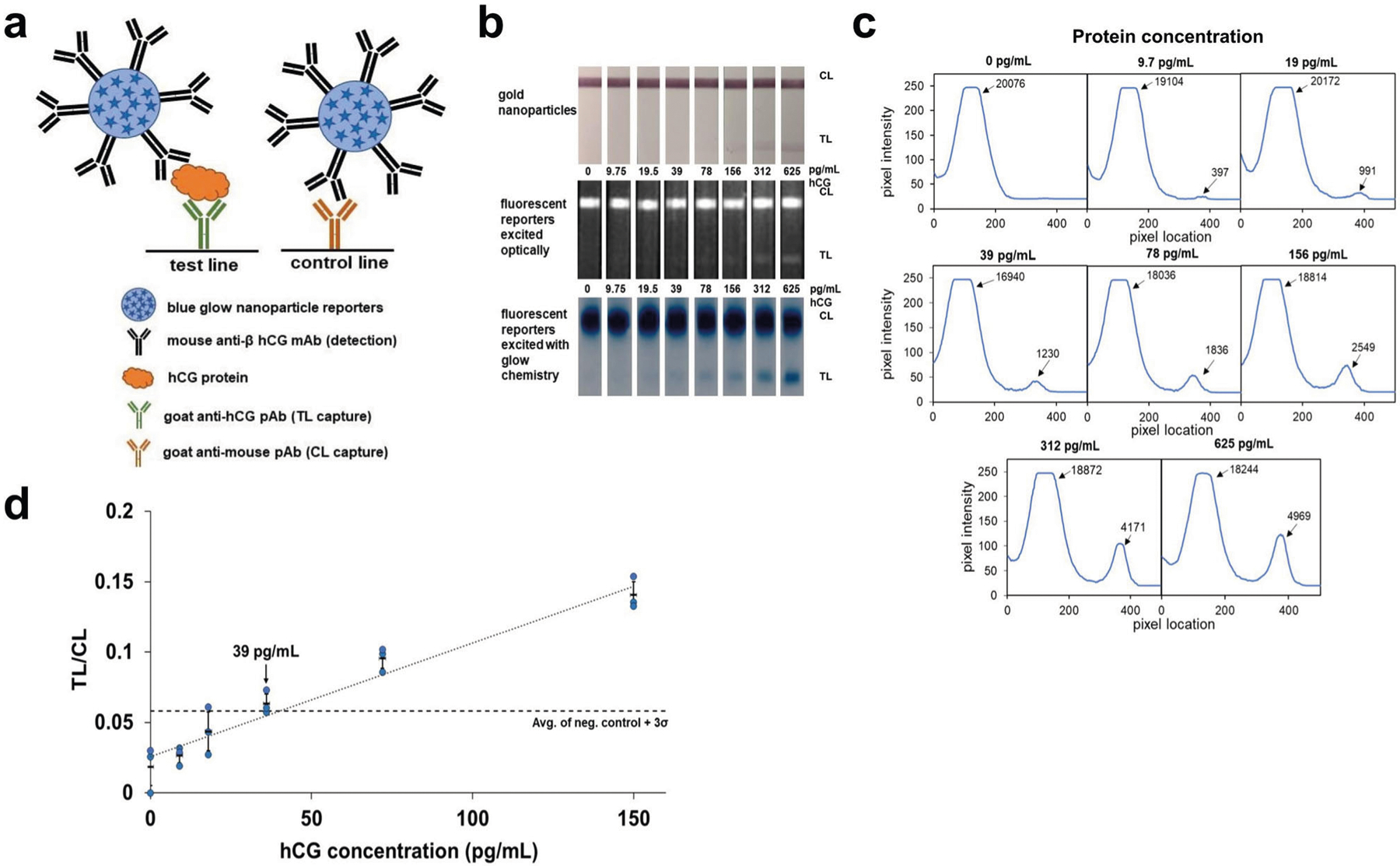
Glow LFA detection of hCG protein. **a** Schematic showing anti-hCG antibody-functionalized glow reporters bound to hCG and captured by anti-hCG polyclonal antibodies on the test line (TL) and unbound glow reporters captured by anti-mouse polyclonal antibodies on the control line (CL). **b** LFA strips for the detection of serially-diluted hCG protein with 40 nm anti-hCG-gold reporters (top) and with anti-hCG blue fluorescent reporters excited using optical excitation (middle) and using glow chemistry (bottom, inverted blue channel image). The LFA strips with blue fluorescent particles were excited optically and imaged using a Fluorchem camera. The same strips were then chemi-excited with glow chemistry and imaged inside a 3D-printed dark box with a Samsung Note 8 camera with an 8-s exposure. Gold LFA strips were imaged with a Samsung Note 8 camera under ambient light. **c** Intensity profiles along the length of the LFA strips shown in (b) were extracted with LFA image analysis software developed in-house; values for the integrated area under the curve for each peak (TL and CL) are shown. **d** Average glow LFA intensity ratios (integrated area ratio: TL/CL) ± 1 Std Dev at different hCG concentrations (*n* = 3); the blue circles represent the individual value for each replicate. The dashed line denotes the average of blank + 3 Std Dev of blank and the dotted line denotes the linear correlation from 0 pg/mL to 156 pg/mL (*R*^2^ = 0.96). The limit of detection is estimated to be 39 pg/mL hCG and the limit of quantitation is estimated to be 156 pg/mL hCG.

In-house dyed polystyrene particles (blue; 9,10-diphenylanthracene) were functionalized with mouse anti-β hCG antibodies and run with varying concentrations of hCG on nitrocellulose anti-hCG half-strips (Fig. 3a) (see Methods). The strips were first optically excited and imaged using a FluorChem system (Fig. 3b, middle) and images analyzed with NIH ImageJ^38^. Intensity analysis of the optically-excited strips detects a test line at 39 pg/mL above background, with no test lines visible at lower concentrations (Supplementary Fig. 5). The same strips were then chemi-excited, imaged using a Samsung Note 8 smartphone camera, and analyzed with in-house analysis software (freely available from our GitHub account, https://github.com/willsonlab/LFA-Image-Analysis^39^). We achieved an LoD of 39 pg/mL hCG (the lowest concentration tested with a signal above the chosen cutoff, the no-target negative control (blank) +3 Std Dev of blank)), with a faint test line visible even at 19.5 pg/mL (Fig. 3b–d), ten times more sensitive than an in-house prepared gold LFA using standard 40-nm gold nanoparticles and the same antibodies (Fig. 3b), and as sensitive as the (optically excited; Supplementary Fig. 5) fluorescent reporters albeit without requiring a filter-based imaging lab instrument. As shown in Fig. 3b, the lines in glow LFAs are typically wider than those of gold LFAs, especially in high-intensity lines. We hypothesize that these wider lines arise from dispersion of light to the surrounding environment, and possibly from dye leaching upon mixing of the particles with the solvents in the excitation solution.

To assess the compatibility of glow chemi-excitation with clinically-relevant samples, blue ThermoFisher FluoSpheres were chemi-excited in the presence of various biological matrices and were imaged with the smartphone camera. Glow brightness in 25% human blood serum was 95% of that in buffer, and it was 70% in nasal swab extract, and 88% in 25% saliva, suggesting compatibility of glow chemistry with multiple biological matrices (Supplementary Fig. 6).

Moreover, to test glow LFA performance in a clinically-relevant matrix, we spiked 1.2 ng/mL of hCG in human serum diluted with LFA running buffer to various extents. After running the strips and before chemi-excitation, we analyzed the optically-excited fluorescence signals of the strips. The LFA strips were then chemi-excited with 30 μL of glow excitation solution and imaged with the Samsung Note 8 smartphone camera. Both the optically-excited fluorescence image (independent of glow excitation) and the glow smartphone image showed that in 100% serum, the particles failed to migrate through the strip and produced no detectable test line (TL) or control line signal. In 75% serum, the TL glow signal was 70% of that in buffer (Supplementary Fig. 7). The optical fluorescence image also showed a reduced TL signal in 75% serum, suggesting that most of the reduction in glow signal was not due to the inhibition of glow excitation by serum. As noted above there was only modest inhibition of glow signal by 50% serum (Supplementary Fig. 6) indicating that the low LFA TL signal was probably due to matrix interference with particle flow and/or antibody binding. This can be mediated through further serum dilution, buffer and sample prep optimization, and/or LFA materials optimization.

### Ultrasensitive SARS-CoV-2 nucleoprotein detection using glow LFA.

**Fig. 4:**
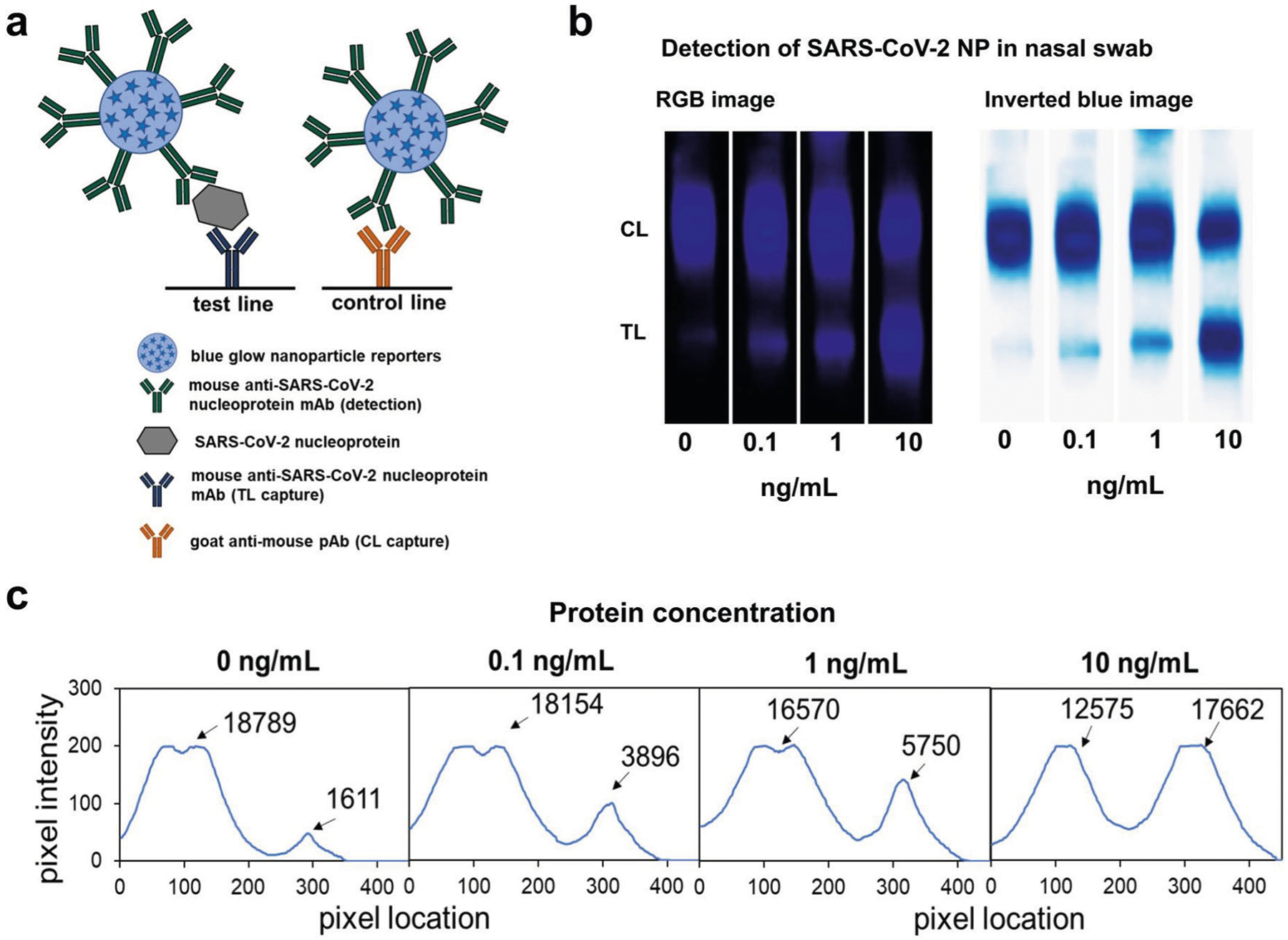
Detection of SARS-CoV-2 nucleoprotein spiked in nasal swab extract using glow LFA. **a** Schematic showing anti-SARS-CoV-2 nucleoprotein (NP) antibody-functionalized glow reporters bound to SARS-CoV-2 NP and captured by anti-SARS-CoV-2 NP antibodies on the test line and unbound glow reporters captured by anti-mouse polyclonal antibodies on the control line. **b** RGB and inverted blue channel images showing glow LFA detection of 100 pg/mL SARS-Cov-2 NP spiked into nasal swab extract (in lysis buffer). Strips were imaged inside a 3D-printed dark box with a Samsung Note 8 camera. **c** Intensity profiles along the lengths of LFA strips shown in (**b**); calculated areas under the curve for each peak are shown.

Nanoparticle-based lateral flow assays have been extensively explored in the detection of infectious diseases and more recently in the development of COVID-19 rapid diagnostics^40–43^. For the initial demonstration of the chemi-excited glow LFA in clinically relevant applications, we developed a glow LFA to detect SARS-CoV-2 nucleoprotein in nasal swab extract. We chose two high-performing anti-SARS-CoV-2 nucleoprotein antibodies identified by ref.^44^ as our LFA detection and capture antibodies. Recombinant SARS-CoV-2 nucleoprotein (Acro Biosystems; #NUN-C5227) was diluted into fresh nasal swab extract negative for COVID-19, run on LFA strips, and chemi-excited (see Methods). 100 pg/mL of SARS-CoV-2 nucleoprotein spiked into nasal swab extract was readily detected above background (nasal swab extract negative for COVID-19) (Fig. 4). While LFA optimization is needed to reduce non-specific binding in the no-analyte test line; preliminary performance is already comparable to those of recently-reported fluorescent LFAs read by specialized readers^45,46^.

### Glow color multiplexing.

We demonstrated simple multiplexing of glow particles using red (Excitation 580 nm/Emission 605 nm), green (Excitation 505 nm/Emission 515 nm), and blue (Excitation 365 nm/Emission 415 nm) ThermoFisher FluoSpheres mixed in different ratios, chemi-excited, and imaged using a smartphone camera. Multiplexing potential was assessed using red, green, and blue imaging channels to assess inter-channel bleed.

**Fig. 5:**
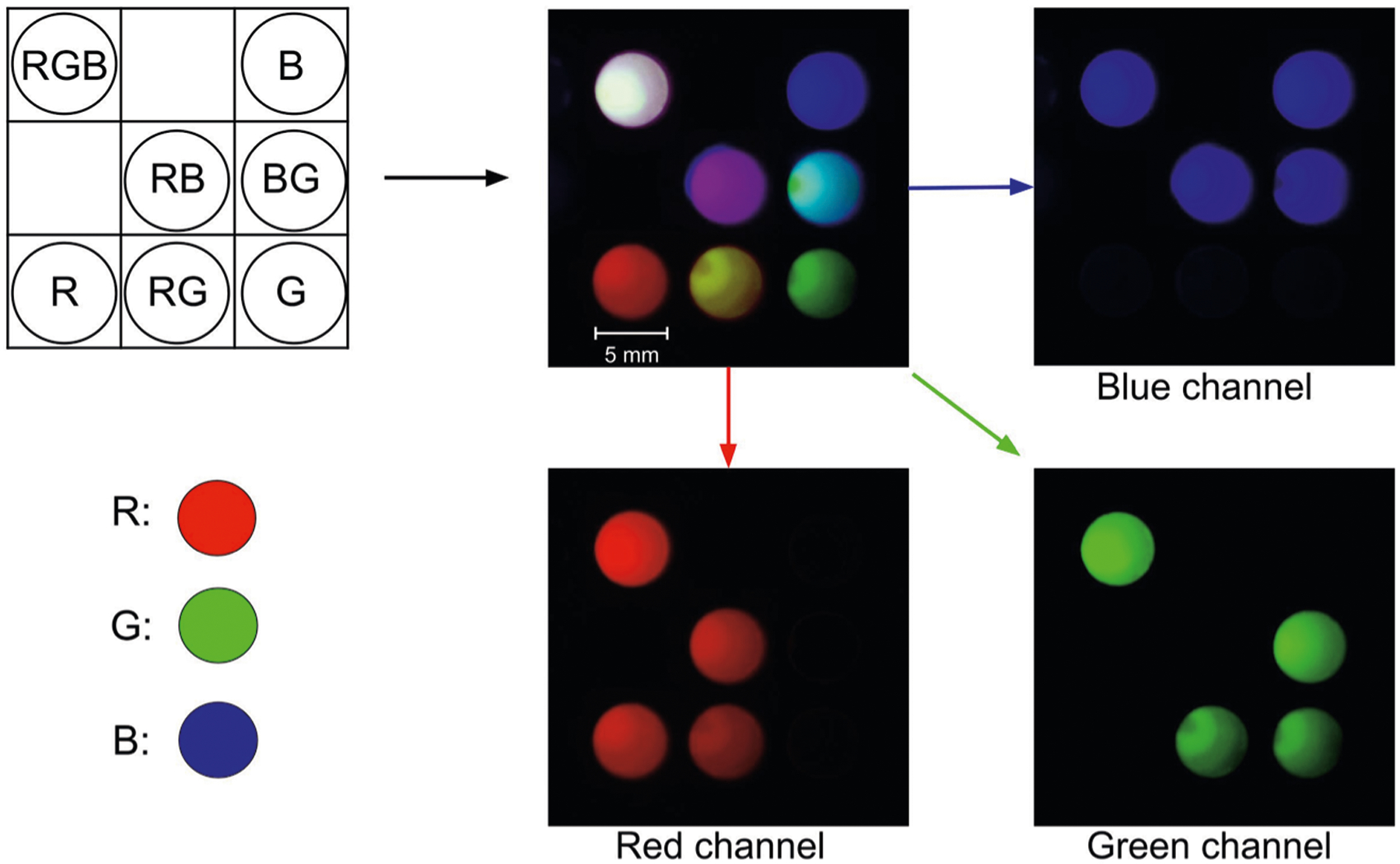
Multiplex detection of glow particles of different colors. FluoSpheres Microspheres red (R), green (G), and blue (B) particles were mixed in various combinations in the wells (5 mm) of a 96-well microplate, chemi-excited with glow excitation solution and imaged with a Samsung Note 8 camera. RGB channels were color-split using ImageJ to assess channel bleed and assay multiplexing potential. Sample wells with single-color particles (R, G, B) had 10 μL of 1 × 10^9^ particles/mL of each color in butyl benzoate. Sample wells with binary mixtures (RB, BG, RG) had 5 μL of each color at 1 × 10^9^ particles/mL. The ternary mixture (RGB) was prepared at a 1:1:1 ratio with 3.3 μL of 1 × 10^9^ particles/mL of each color.

We confirmed that all particles were effectively chemi-excited with a single glow reagent and imaged using a smartphone camera (Fig. 5). Despite the inherently non-uniform spectral response of the smartphone camera^47,48^ and the broad emission spectra of specific fluorophores, this specific selection of dyes with appropriate emission spectra gave no significant inter-channel signal bleed when the exposure time was kept below the camera sensor saturation level.

**Fig. 6:**
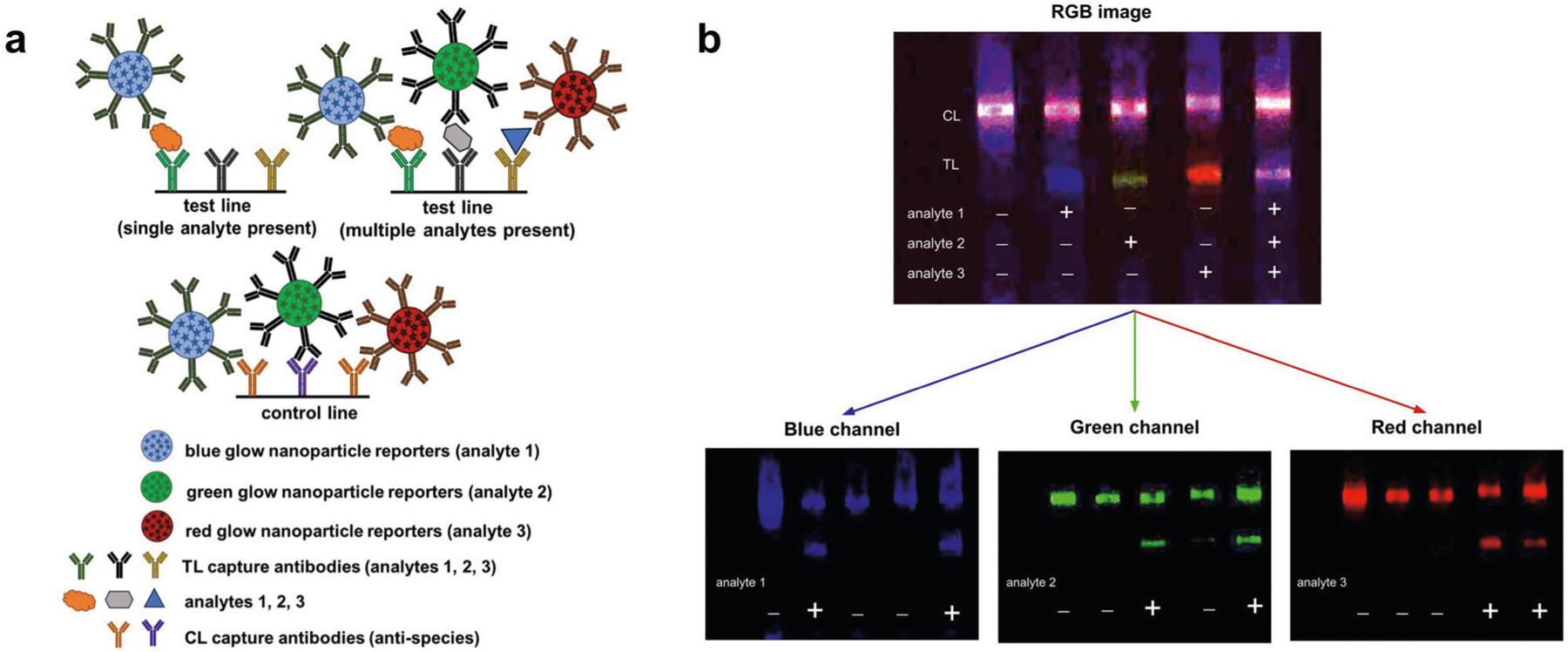
Color multiplex detection of three analytes on a single line using glow LFA. **a** Schematic showing red, blue, and green glow reporters functionalized with antibodies specific to separate target analytes. The LFA test lines contain a mixture of three capture antibodies specific to the three analytes. When a single analyte is present, only the reporter particle specific to that analyte will bind to the test line, creating a single-color test line. When more than one analyte is present, multiple particles will bind to the test line creating a multicolor test line. **b** To demonstrate the feasibility of a glow multiplex LFA, three different color glow particles specific to different analytes were mixed together and run on a multiplex LFA strip. When only analyte 1 is present a blue test line will form, when only analyte 2 is present a green test line is formed, when only analyte 3 is present a red test line will form, and when all three analytes are present a multicolor test line will form. The RGB image is shown at the top of **b**, and the individual channels (blue, green, red) are shown at the bottom. The channels were split using ImageJ. This channel deconvolution can be used to distinguish the presence of each single analyte on a single multi-antibody test line.

We further demonstrated the feasibility of multiplexing glow LFAs by detecting three analytes simultaneously on a single test line, recently described “as probably the most powerful method of multiplexing in LFAs*”*^49^. Blue, red and green glow particles were functionalized with three different detection antibodies, each one specific to a separate target analyte (see Methods). Multiplex LFA strips were constructed by striping a test line containing a mixture of three different capture antibodies (one antibody specific to each analyte) and a control line containing a mixture of anti-species antibodies corresponding to the species of the detection antibodies. To perform the multiplex LFA, all three particles were mixed together and added to the LFA running buffer containing only one of the analytes (1, 2, or 3) or a mixture of all three analytes (Fig. 6a). The LFA strips were then chemi-excited with 30 μL of glow excitation solution and imaged with the Samsung Note 8 smartphone camera. In the presence of a single analyte (Fig. 6b), the test line will appear as a single color and the control line will appear as a mixed color (red, green, and blue particles are captured on the control line); in the presence of all three analytes, the test line will also appear as a mixed color. The test line signal can be deconvoluted by standard RGB channel separation to determine which analytes are present—blue channel signal denotes the presence of analyte 1, if a signal shows in the green channel analyte 2 is present, and a red channel signal shows that analyte 3 is present. Our initial demonstration shows that when all three analytes are present together in the sample, the presence of each analyte can be confirmed using channel separation, with minimal bleed-over from each particle into other channels, confirmed with intensity analysis of each channel for each strip (Supplementary Fig. 8). Signal deconvolution could readily be integrated into a smartphone application, allowing for smartphone LFA detection and analysis of multiple analytes simultaneously (three analytes if using one test line, 6 analytes if using two test lines, etc., assuming the available antibodies have minimal cross-reactivity).

## Discussion

We have demonstrated a new approach to sensitive, smartphone-readable, and multiplexable LFAs using chemical excitation of fluorescent LFA reporters. We have demonstrated sensitive glow LFAs for the detection of hCG (LoD of 39 pg/mL (1 pM)) and for SARS-CoV-2 nucleoprotein (100 pg/mL (2.1 pM) in nasal extract), a performance that rivals other next-generation LFAs based on e.g., fluorescence, and even ELISA, the gold standard for immunoassays^50^. Our glow LFA format resembles the conventional enzymatic-based LFA workflow but without requiring the use of fragile enzymes or expensive and temperature-sensitive light-generating chemiluminescent substrates, and does not require the expensive or complex optics used for fluorescent LFAs. In addition to single-analyte detection with a single-color reporter, we also demonstrated the detection of multiple analytes on a single LFA test line using three different-colored glow particles, a single common chemi-excitation reagent, and a standard smartphone camera. We currently use glass ampoules containing oxalate and peroxide enclosed in a plastic dropper, and activate the reagents by bending to break the ampoules (like a glowstick) and then invert the dropper a few times to mix the reagents before applying the mixture onto the LFA strip. Our eventual goal is to store reagents in blisters built into the cassette and delivered to the LFA strip with a push, limiting user exposure to reagents and simplifying assay workflow. Although peroxyoxalate chemi-excitation has been used previously in flow injection analysis^51^, in conjunction with nanomaterials (anatase^52^ and mesoporous silica nanoparticles^53^), with polymer-based oxalate esters^51^, and in ELISA^54,55^, it has not to our knowledge been applied or adapted for a POC diagnostic format such as LFA. Unlike other high-sensitivity LFA reporter technologies, glow LFA increases the sensitivity of analyte detection without compromising simplicity and POC applicability.

## Methods

### Fluorescent nanoparticles.

FluoSpheres Carboxylate-Modified Microspheres: red (0.2 μm, Excitation 580 nm/Emission 605 nm; #F8763), green (0.2 μm, Excitation 540 nm/Emission 560 nm; #F8809), yellow-green (0.2 μm, Excitation 505 nm/Emission 515 nm; #F8888), blue (0.2 μm, Excitation 365 nm/ Emission 415 nm; #F8805), and red Fluoromax polystyrene (unmodified) (0.3 μm, Excitation 542 nm/Emission 612 nm; #R300), were purchased from ThermoFisher. Non-functionalized polystyrene particles (288 nm, Bangs Laboratories; #PS02009) were dyed in-house by combining 600 μL of particles at 1% solids in water and 100 μL of 15% tetrahydrofuran (THF) (Millipore Sigma; #401757) containing 5 mM 9,10-diphenylanthracene (Sigma Aldrich; #D205001–1G-A). The particles and dye were incubated at room temperature on a rotator for 1 h, then washed 10× with water by centrifugation for 10 min at 16,500 × *g*, removing the supernatant and re-suspending the pellet in water.

### Glow excitation solution.

We screened various bis(2,4,6-trichlorophenyl) oxalate (TCPO) solvents (ethyl acetate, tributyl O-acetylcitrate, butyl benzoate, tributyl citrate) and cosolvents (containing H_2_O_2_; tert-butanol, methanol) to minimize toxicity and odor and maximize glow signal strength and stability (Supplementary Table 1). The final formulation of oxalate substrate contained 15 mM TCPO (Millipore Sigma; #O3629) dissolved in 33.3% butyl benzoate (Millipore Sigma; #293296) and 66.6% tributyl citrate (Millipore Sigma; #27497), mixed on a rotator at room temperature for at least 5 h until fully dissolved by visual inspection. The final formulation of the H_2_O_2_ substrate contained 45 mL of tert-butanol (Sigma Aldrich; #471712), 5 mL of 30% H_2_O_2_ (3% final), and 1 mM tetrabutylammonium hydrogen sulfate, a base catalyst (Millipore Sigma; #155837). During screening, the TCPO and H_2_O_2_ substrates were mixed at a 1:1 ratio and (within 5 min) added to glow particles (in a well) or pipetted onto a glow LFA strip. No observable loss of excitation potency was observed for at least 5 min with all three colors of particles after mixing. We typically image the LFA strips within 60 s of addition of the glow excitation solution.

### Optical and chemical excitation of 9,10-diphenylanthracene.

9,10-diphenylanthracene (Sigma Aldrich; #D205001–1G-A) was diluted to 50 mM in tetrahydrofuran (Sigma Aldrich; #D205001–1G-A) and further serially diluted in butyl benzoate. 50 μL of the dye serial dilutions were pipetted into the wells of a white (chemi-excitation) or black (optical excitation) 96-well plate. The dye was then optically excited and emitted light was measured in a Tecan M200PRO microplate with Excitation 365 nm/Emission 415 nm. The dye was chemi-excited with glow excitation solution (50 μL) and imaged with a smartphone. Intensity profiles for chemi-excited particles were extracted from the smartphone images using NIH ImageJ as described in Methods.

### Optically- and chemically-excited emission spectra and detectability of fluorescent particles.

All optically-excited experiments were done with particles diluted in 50 μL butyl benzoate in a 96-well black polystyrene plate (Corning; #3991) and measured with the Tecan M200PRO microplate reader by their corresponding excitation and emission wavelengths (in-house particles with 9,10-diphenylanthracene dye: Excitation 350 nm/Emission 425 nm; Thermo Fisher FluoSpheres; #F8805: Excitation 365 nm/Emission 415 nm; Thermo Fisher FluoSpheres #F8888: Excitation 505 nm/Emission 515 nm; Thermo Fisher FluoSpheres #F8763: Excitation 580 nm/Emission 605 nm).

All chemi-excited experiments were done by mixing particles in butyl benzoate with 50 μL of glow excitation liquid in a 96-well white polypropylene plate (Corning; #3355). Chemi-excitation emission spectra were obtained using a Tecan M200PRO microplate reader with excitation set at 850 nm (outside the instrument reading range) to scan particles’ emission spectra. Smartphone readings were imaged with a Samsung Note 8 camera and a 3D-printed dark box using the built-in Samsung camera Pro mode with an exposure time of 10 s (max), white balance set to 5500 K, and ISO set to 800 (max), and the image was analyzed using ImageJ.

The detectability of optically-excited fluorescent particles with the Tecan M200PRO plate reader and of chemi-excited fluorescent particles with a Samsung smartphone was performed by (two-fold) serially diluting FluoSpheres Microspheres (blue, green, and red particles) starting with 2 × 10^8^ particles/well. The two dilution series were read with the Tecan plate reader and with the Samsung smartphone according to the methods described above. The intensity values for the chemi-excited particles were normalized to their respective no-particle control values.

### Glow signal stability.

Red particles (0.3 μm, Excitation 542 nm/Emission 612 nm; #R300), green particles (0.2 μm, Excitation 540 nm/Emission 560 nm; #F8809), and blue particles (0.2 μm, Excitation 365 nm/Emission 415 nm; #F8805) were pipetted into the wells of a white 96-well plate (Corning; #3916) (2 × 10^9^ particles per well; three replicates per sample). 100 μL of a 1:1 mixture of 15 mM TCPO and 3% H_2_O_2_ (glow excitation solution) was added to each well and mixed thoroughly. The plate was placed inside a 3D-printed dark box, imaged with a Samsung Note 8 camera (4 s exposure, white balance 5500 K, ISO 400), and imaged every minute for 10 min. The images were analyzed using ImageJ (ROI manager). Intensity values were averaged, then normalized to the maximum value for each color particle.

### Particle conjugation with anti-hCG antibodies.

The in-house dyed polystyrene particles described above were functionalized with antibodies by passive adsorption. Briefly, 35 μg (35 μL of 1 mg/mL) of mouse anti-β hCG antibody (Arista Biologics; clone 2; #CGBCG-0402) in 1 × PBS (pH 7.4) (Takara; #T9183) was added to 100 μL particles (0.5% w/v) and incubated at 20 °C for 3 h on a benchtop rotator at 5 rpm. The particles were centrifuged for 10 min at 16,500 × *g*, resus-pended in 1 mL 1% BSA (Sigma Aldrich; #A7906) in 1× PBS, and placed on a tube rotator overnight at 4 °C. After blocking, particles were washed 10 times by resuspension and centrifugation (10 min at 16,500 × *g*) into 1% BSA in 1× PBS. After washing, the functionalized particles were stored at 0.5% solids in 1 × PBS, 1% BSA at 4 °C.

Gold nanoparticles were functionalized by adding 100 μl of 4 mM KCl to 1 mL of stock particles (DCN; #CG, 40-nm gold nanoparticles supplied at 1 OD) in a Protein LoBind Tube (Eppendorf; #0030108442). Then, 10 μg of mouse anti-β hCG antibody (Arista Biologics; clone 2; #CGBCG-0402) in 1× PBS (pH 7.4) (Takara; #T9183) was added for 30 min at 20 °C on a benchtop rotator at 5 rpm. BSA, 100 μL (10% (w/v)), was then added to block the nanoparticles and placed on a tube rotator for 20 min. After blocking, the gold nanoparticles were collected by centrifugation (10 min, 10,000 × *g*) and washed once by resuspension in 1 mL of storage solution (PBS, pH 7.4, 1% (w/v) BSA, 10% (w/v) sucrose) and centrifugation (10 min, 10,000 × *g*). The functionalized particles were stored in 100 μL of storage solution at 4 °C. To estimate the concentration of gold nanoparticles, their absorbance at 520 nm was measured and compared to the stock (particles/mL = 10^11^ × OD520).

### Commercial fluorescent particle conjugation with anti-SARS-CoV-2 NP antibodies.

Carboxylate-modified fluorescent particles (ThermoFisher Fluorospheres; #F8805) were functionalized with mouse anti-SARS-CoV-2 nucleoprotein monoclonal antibodies (SinoBiological; #40143-MM08) using standard EDC-NHS chemical activation. Briefly, 100 μL particles at 0.5% solids were centrifuged (10 min at 16,500 × *g*) and washed twice with 50 mM MES buffer, pH 5.8. The particles were resuspended in 95 μL MES buffer and sonicated until no visible aggregates remained. Particles were activated by EDC (ThermoFisher; #A35391) and NHS (Millipore Sigma; #130672) at a molar ratio of NHS:carboxyl groups of 20 (3.4 μL of 50 mg/mL NHS) and EDC:carboxyl groups of 2.5 (3.5 μL of 10 mg/mL EDC). NHS and EDC were added to the resuspended particle mixture sequentially (NHS followed by EDC) and placed on a benchtop rotator for 30 min at 20 °C. After activation, the particles were washed twice by centrifugation in 1 × PBS and resuspended by sonication. 35 μg of antibody (35 μL of 1 mg/mL suspended in 1× PBS) was added to the particles and incubated at 20 °C on a benchtop rotator for 2 h. The mixture of particles and antibody was centrifuged (10 min at 16,500 × *g*), the supernatant was removed, and particles were resuspended in 4% BSA in 1× PBS and incubated for 1 h at 20 °C on a benchtop rotator. The particles were washed three times by centrifugation with 1 × PBS, 1% BSA solution, resuspended at 0.5% solids in 100 μL 1× PBS, 1% BSA, and stored at 4 °C.

### Conjugation of commercial fluorescent particles with anti-hCG antibodies, anti-SARS-CoV-2 NP antibodies, and anti-PSA antibodies for glow multiplex LFA.

Commercial blue carboxylate-modified fluorescent particles (ThermoFisher; #F8805) were functionalized with mouse anti-β hCG antibody (Arista Biologics; clone 2; #CGBCG-0402) and commercial green carboxylate-modified fluorescent particles (ThermoFisher; #F8809) were functionalized with rabbit anti-SARS-CoV-2 nucleoprotein (ExonBio; #NP11H9) (as described above in Methods—Commercial fluorescent particle conjugation with anti-SARS-CoV-2 NP antibodies). Red unmodified fluorescent particles (ThermoFisher; #R300) were functionalized with mouse anti-prostate specific antigen-antibody (Abcam; #ab403) with passive adsorption (as described above in Methods—Particle conjugation with anti-hCG antibodies). All particles were stored at 0.5% solids in 100 μL 1× PBS, 1% BSA at 4°C.

### Human chorionic gonadotropin (hCG) LFA strip preparation.

The architecture of a typical LFA strip and the process of making LFA strips in our lab are shown in Supplementary Fig. 1. FF80HP nitrocellulose membrane (Cytiva; #13549206) was cut to 2.5 cm using a craft paper cutter (Fiskars; #199080–1007) and assembled on a 30-cm backing adhesive card (DCN Diagnostics; #MIBA-020) along with a CF5 absorbent pad (Cytiva; #8115–2250). The assembled membrane was striped using a BioDot dispenser (BioDot; #XYZ30600124), at a flow rate of 1 μL/cm and a dispensed volume of 30 μL per 30 cm card. The test line contained goat polyclonal anti-αhCG antibodies (Arista; #ABACG-0500) diluted to a concentration of 1 mg/mL in 1 × PBS. The control line contained goat polyclonal anti-mouse antibodies (Arista; #ABGAM-0500) diluted to a concentration of 1 mg/mL in 1 × PBS. Striped membranes were dried in a Robbins Scientific Micro Hybridization Incubator 2000 at 37 °C for 30 min, then stored overnight at 20 °C in a desiccator chamber (Totech; SuperDry Desiccant Cabinet; #SD-151–21) at 5% humidity. The striped card was cut into 3 mm strips using a KinBio ZQ2000 Guillotine Cutter and stored at 20 °C in sealed 50 mL conical tubes (USA Scientific; #5622–7261) with desiccant packs (Interteck Packaging; #IN1G51).

### SARS-CoV-2 LFA strip preparation.

FF120HP nitrocellulose membrane (Cytiva; #13549205) was cut to 2.5 cm using a craft paper cutter and assembled onto a backing adhesive (DCN Diagnostics; #MIBA-020) with a CF5 absorbent pad (Cytiva; #8115–2250) and a Standard 14 sample pad (Cytiva; # 8133–2250). The assembled, uncut membrane was stripped using a BioDot dispenser (BioDot; #XYZ30600124), with a flow rate of 1 μL/cm and a dispense volume of 30 μL per 30 cm card. Striped membranes were dried in a Robbins Scientific Micro Hybridization Incubator 2000 at 37 °C for 30 min, then stored overnight at 20 °C in a desiccator chamber (Totech; SuperDry Desiccant Cabinet; #SD-151–21) at 5% humidity to dry completely. The striped card was then cut into 3 mm strips using a KinBio ZQ2000 Guillotine Cutter and stored at 20 °C in sealed 50 mL conical tubes (USA Scientific; #5622–7261) with desiccant packs (Interteck Packaging; #IN1G51). The test lines for each set of strips contained mouse anti-SARS-CoV-2 antibodies (Bioss; #bsm-41411M) at 1 mg/mL in 1× PBS, and the control lines contained goat polyclonal anti-mouse antibodies (Arista; #ABGAM-0500) at 1 mg/mL in 1× PBS.

### LFA running protocol for hCG and SARS-CoV-2 LFA.

The LFA running and wash buffer for the hCG test (LFA buffer A) contained 1× PBS (pH 7.4), 0.5% BSA (Millipore Sigma; #A9418), 0.5% Tween-20 (Millipore Sigma; #P1379), and 0.3% PEG 3000 (Millipore Sigma; #81227). hCG (Millipore Sigma; #CG10) was diluted in LFA buffer A or in serum pre-diluted with LFA buffer A. LFA buffer A was also used as a diluent for the determination of SARS-CoV-2 nucleoprotein (Acro Biosystems; #NUN-C5227) limit of detection in buffer. The LFA running buffer (and nasal swab extraction buffer) for detection of SARS-CoV-2 nucleoprotein spiked into nasal swab extract (LFA buffer B) contained 1× PBS (pH 7.4), 1% BSA (Millipore Sigma; #A9418), 0.5% Tween-20 (Millipore Sigma; #P1379), and 0.1% IGEPAL CA-630 (Millipore Sigma; #I8896).

LFAs for hCG and SARS-CoV-2 were run in half-strips consisting only of membrane and absorbent pad. Half-strips were submerged in a 2 mL conical base tube (USA Scientific; #1420–2700) containing 30 μL of a solution containing the analyte and 5 × 10^8^ particles glow reporter nanoparticles (or anti-hCG gold nanoparticles, 1 × 10^9^ particles per sample) in LFA buffer A. LFAs for serum samples and nasal swab extracts also included Cytiva Standard 14 sample pads, upon which 30 μL of the sample was pipetted. After the sample had wicked through the membrane completely (about 15 min), each LFA strip was washed with 30 μL of wash buffer (LFA buffer A for hCG detection in buffer and serum; LFA buffer B for SARS-CoV-2 nucleoprotein detection in nasal swab extracts) by either dipping (half-strips) or pipetting wash buffer onto the sample pad.

### Multiplex LFA strip preparation and running protocol.

LFA strips were assembled, striped and cut as described above for the SARS-CoV-2 nucleoprotein glow LFAs using CN95 membrane (Sartorius; #1UN95ER100025NTB) and 8980 as a sample pad (Ahlstrom; #8980). However, for multiplex LFA strips, the test line contained a mixture of 3 antibodies: *antibody 1*: goat polyclonal anti-hCG-α antibodies (Arista; #ABACG-0500) at 1.0 mg/mL in 1× PBS, *antibody 2*: mouse anti-SARS-CoV-2 antibodies (Bioss; #bsm-41411M) at 1.0 mg/mL in 1× PBS and *antibody 3*: goat polyclonal anti-PSA antibodies (R&D systems; AF1344) at 0.3 mg/mL in 1× PBS. The control line contained a mixture of goat polyclonal anti-mouse antibodies (Arista; #ABGAM-0500) at 0.5 mg/mL and goat polyclonal anti-rabbit antibodies (Arista; #ABGAR-0500) at 0.5 mg/mL in 1× PBS. The corresponding model analytes used were: *analyte 1*: human chorionic gonadotropin (hCG; Millipore Sigma; #CG10), *analyte 2*: SARS-CoV2 nucleoprotein (Acro Biosystems; # NUN-C5227) and *analyte 3*: prostate-specific antigen (PSA; Sigma; #P3235–10UG). For the single-target LFA experiments, analyte 1 was diluted at 5 ng/mL, analyte 2 at 10 ng/mL, and analyte 3 at 100 ng/mL in LFA buffer (1× PBS (pH 7.4), 0.5% BSA (Millipore Sigma; #A9418), 0.5% Tween-20 (Millipore Sigma; #P1379), and 0.3% PEG 3000 (Millipore Sigma; #81227)). For multiplex LFA experiments, the analyte mixture contained analyte 1 at 5 ng/mL, analyte 2 at 10 ng/mL, analyte 3 at 100 ng/mL in LFA buffer. 5 × 10^8^ reporter particles corresponding to each analyte (blue—analyte 1, green—analyte 2, red—analyte 3) were added to single analyte or multiplex analyte mixtures. 30 μL of the analyte/particle mixture was pipetted onto the sample pad. After the sample had wicked through the membrane completely (about 5 min), each LFA strip was washed with 30 μL of wash buffer (LFA buffer A) by pipetting onto the sample pad.

### Strip development, imaging, and analysis.

To read the fluorescence signal of glow LFA strips (before chemi-excitation), strips were imaged on a gel documentation system composed of a FluorChem SP gel cabinet (Alpha Innotech Corp., San Leandro CA) and a CoolSNAP K4 CCD 2048 × 2048-pixel camera (Photometrics, Blaine WA) controlled by Micro-Manager 1.4.22 software (Vale Lab, University of California, San Francisco). The strips were optically excited with the built-in epi-illuminating UV lights and imaged through an originally equipped UV cut-off filter with an exposure time of 1 sec and pixel binning of 4.

To read the signals of the gold LFAs, LFA strips were assessed by naked eye, images were acquired with an iPhone XR, or strips were scanned on an Epson Perfection V600 scanner and intensity data were obtained using ImageJ.

To obtain the glow chemi-excitation signal, 30 μL of glow excitation liquid was dispensed directly over the test and control lines, and the strip was immediately imaged inside a custom 3D-printed dark box (Supplementary Fig. 2) with a Samsung Note 8 smartphone camera. Images were taken using the built-in Samsung camera Pro mode with manually set exposure time (2–10 s), white balance set to 5500 K, ISO set to 800 (maximum), and manually focused.

Images were transferred by Wi-Fi from the smartphone to a PC using Google Photos and analyzed using an in-house developed, platform-independent LFA image analysis software (written in Python 3.6 and available at our GitHub account, https://github.com/willsonlab/LFA-Image-Analysis). Besides the RGB color channel, users are prompted to choose a reporter type (colorimetric or luminescent) to determine the polarity of the signal (inverted or non-inverted, respectively). The region of interest for each strip is automatically detected by the software based on intensity threshold, and manually adjusted if needed. The pixel intensity within each region of interest is averaged across the strip width and plotted, and the area under the curve is calculated for each peak with the peak detection function (find_peaks) in the Python SciPy library56. The baseline was determined using the asymmetric least squares smoothing method of Eilers and Boelens57. To properly identify the test and control line peaks, parameters such as prominence (a find_peaks parameter that measures how much each peak stands out relative to the surrounding baseline), relative peak height (measured as a percentage of its prominence), peak separation (minimum horizontal distance between nearby peaks), and baseline can be manually adjusted to correct for different strip geometries and cameras with different bit-depth and resolution.

### Glow color multiplexing.

FluoSpheres Microspheres (Thermo Fisher), red (R; #F8763), green (G; #F8888), and blue (B; #F8805) particles were mixed in various combinations in the wells of a 96-well microplate to assess color multiplexing using glow technology. Sample wells with single-color particles (R, G,B) had 10 μL of 1 × 10^9^ particles/mL of one color. Sample wells with binary mixtures (RB, BG, RG) were 1:1 mixtures of 5 μL of 1 × 10^9^ particles/mL of each color, and a ternary RGB mixture contained a 1:1:1 mixture (3.3 μL of 1 × 10^9^ particles/mL of each color). Particles were chemi-excited with 50 μL TCPO/H_2_O_2_ (1:1 mixture of 15 mM TCPO in butyl benzoate and 3% H_2_O_2_ in tert-butanol) and imaged with a Samsung Note 8 camera (4 s exposure, white balance 5500 K, ISO 800). RGB channels were color-split using ImageJ to assess channel cross-bleed and multiplexing potential.

### Particle experiments in serum, saliva, and nasal swab extract.

50 μL of sample (mixture of 1× PBS, pH 7.4 with serum or saliva diluted at the desired ratio, or nasal swab extracted in 1 × PBS, or 1× PBS control) was mixed with 1 × 10^9^ blue FluoSpheres (ThermoFisher; #F8805) (5 μL in 1× PBS) and pipetted into wells of a white 96-well plate (Corning; #3916) (three replicates per sample). 50 μL of a 1:1 mixture of 15 mM TCPO and 3% H_2_O_2_ (glow excitation solution) was added to each well and mixed thoroughly. The plate was placed inside a 3D-printed dark box, imaged with a Samsung Note 8 camera (4 s exposure, white balance 5500 K, ISO 50), and the image was analyzed using ImageJ (ROI manager).

### Clinical matrices.

Serum from healthy donors was obtained from Gulf Coast Regional Blood Center, Houston, Texas, and stored at −20 °C until used.

Nasal swab specimens were self-collected by adult lab members (asymptomatic and negative by EUA’d LFA for COVID-19) using a Copan FLOQSwab^®^ (Copan; #519CS01) under a University of Houston IRB-approved protocol (IRB: STUDY00002547). Each swab was extracted in 1 mL of LFA buffer B, and the swab was discarded. The extracts were pooled and used immediately.

Saliva was self-collected from adult group members by spitting into a 50 mL sterile tube (UH IRB: STUDY00002547). Samples were pooled and used without further processing.

### Statistics and reproducibility.

Intensity profiles were extracted from the smartphone or FluorChem images using NIH ImageJ or the *in-house* developed LFA Image Analysis software. The data were further processed and plotted using Microsoft Excel software. Each experiment was repeated at least thrice using distinct samples and the collective data were represented as mean value with standard deviation.

### Reporting summary.

Further information on research design is available in the Nature Portfolio Reporting Summary linked to this article.

## Supplementary Material

Supplemental Information

## Figures and Tables

**Fig. 1:**
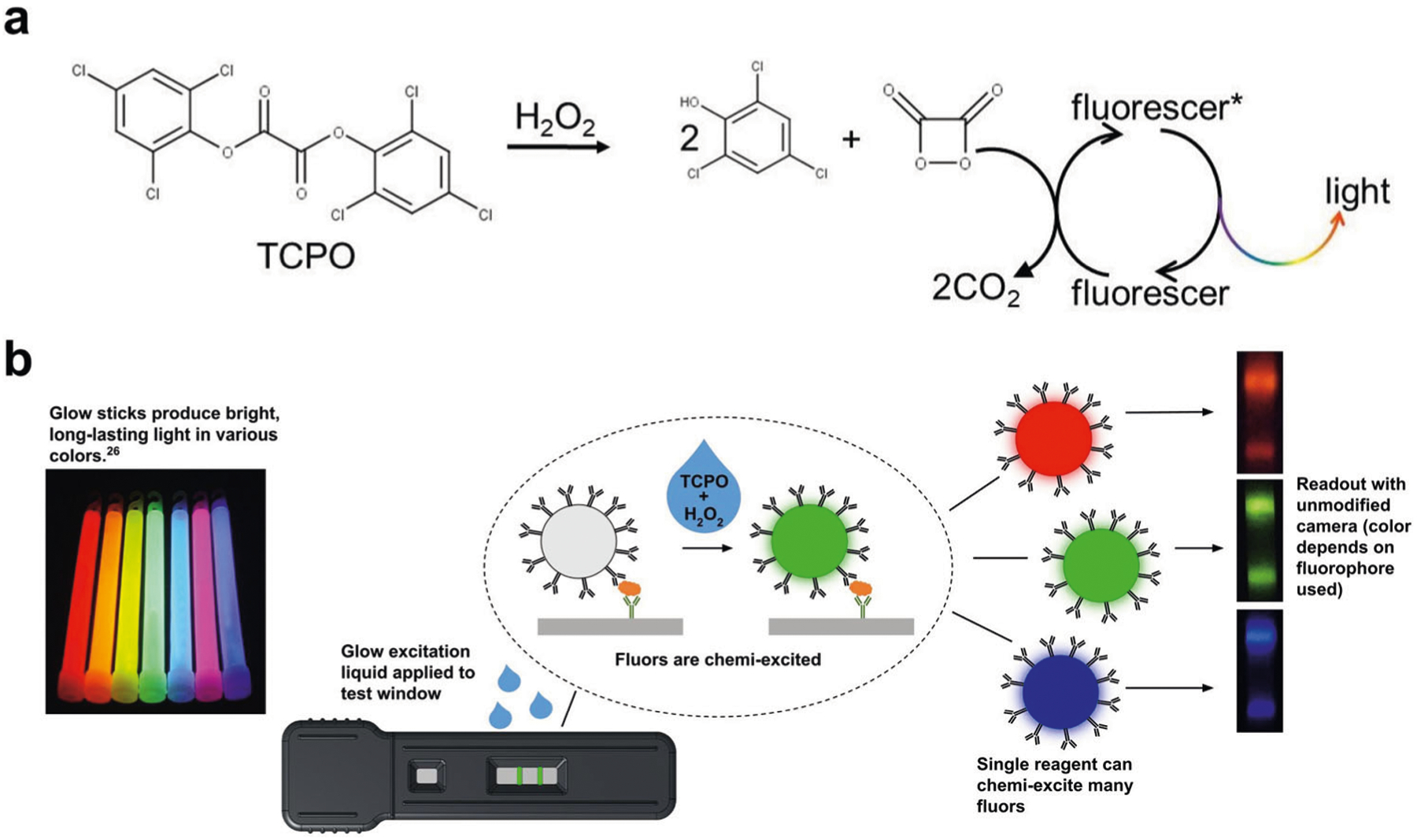
Glow LFA. **a** Mechanism of the TCPO-H_2_O_2_ chemi-excitation glow reaction. TCPO reacts with H_2_O_2_ to generate the energy-rich intermediate 1,2-dioxetanedione, which chemically excites a nearby fluorophore that emits visible light upon relaxation. **b** Application of glow chemistry to LFA. LFA involves capillary wicking of a sample along a dry, porous membrane to a line bearing antibodies specific to a target analyte. In the presence of the target analyte, reporter particles bearing analyte-specific detector antibodies are sandwich-bridged to membrane-bound capture antibodies and accumulate in a line that indicates a positive test. Glow stick chemi-excitation reagents make the particles glow in a highly-detectable way.

## Data Availability

The data supporting the findings of this work are available within the article and its Supplementary Information. Source data underlying the graphs presented in the main and supplementary figures are included in “Supplementary Data 1”.
