# Supplementary material for: Glowstick-inspired smartphone-readable reporters for sensitive, multiplexed lateral flow immunoassays
Source: Commun Eng. Author manuscript; Available in PMC 2024 Apr 5. (PMC10955955; doi:10.1038/s44172-023-00075-2)
Supplement: Supplemental Information [file NIHMS1934145-supplement-Supplemental_Information.pdf]

Supplementary Information

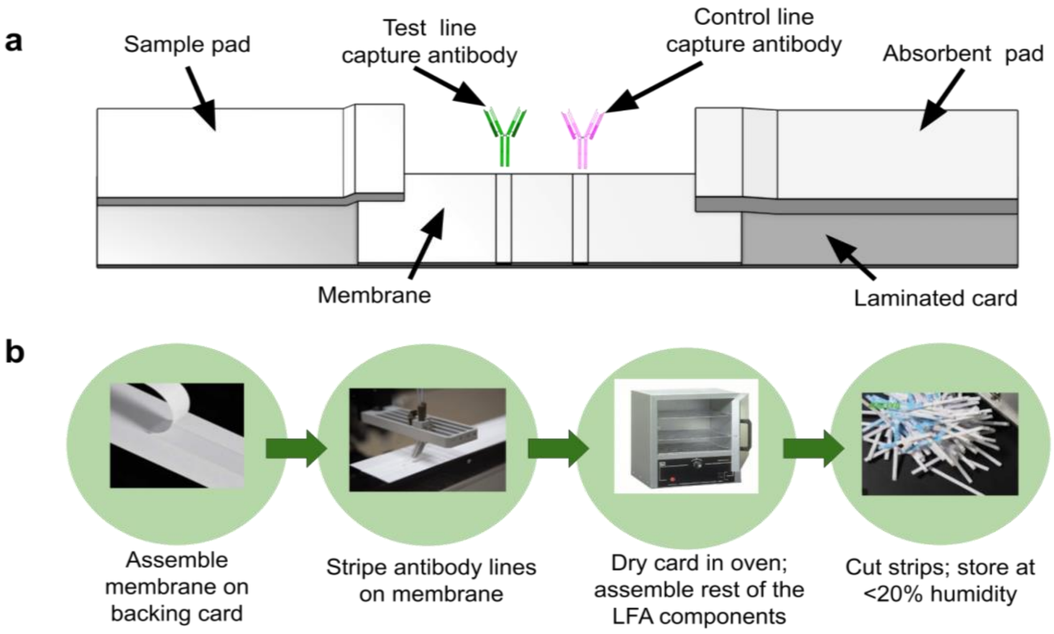

Supplementary Figure 1. Typical preparation of LFA strips in our lab. a Standard LFA components consist of a sample pad, a conjugate pad containing immobilized detection particles (not used here), nitrocellulose membrane containing the test line and control line, and an absorbent pad, all of which are assembled on an adhesive backing card. **b** The nitrocellulose membrane, is assembled onto an adhesive backing card. Antibodies are then deposited onto the membrane using an automated liquid dispensing machine (BioDot XYZ3060) to make the test and control lines. After dispensing, the card is dried in a circulating air oven at a temperature of 37°C or higher for at least one hour. Once the card is dry, other components, such as sample pad (pretreated if required), conjugate pad (sprayed with reporter particles) and absorbent pad are assembled onto the card, the card is cut to the desired width strips using a guillotine and strips are stored at low humidity (<20°C) until ready to use.

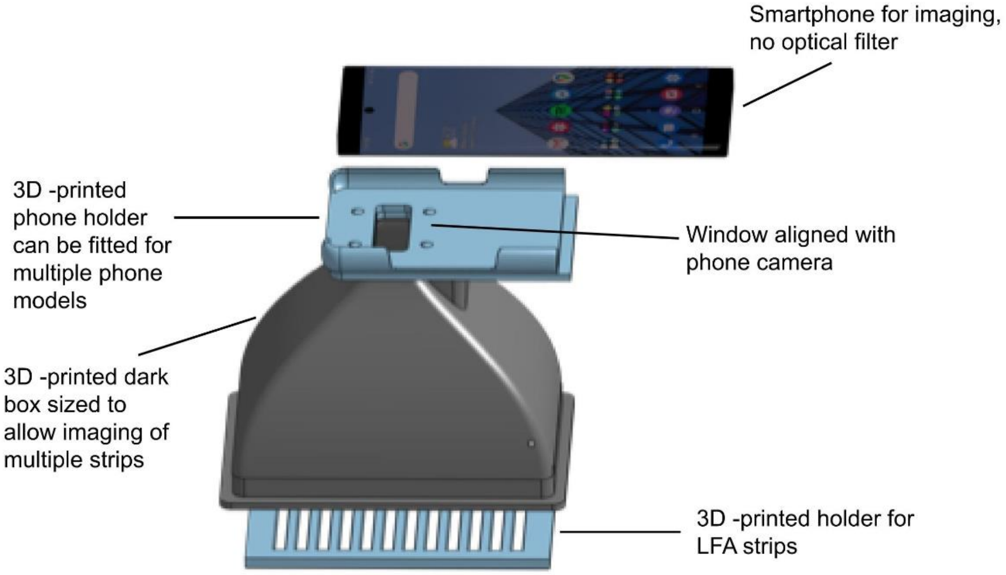

Supplementary Figure 2. Schematic of 3D-printed dark box for imaging. The 3D-printed dark box creates a dark environment that allows for imaging of LFA strips with a smartphone, without the use of optical filters or other expensive equipment. The dark box is sized to allow imaging of up to 12 LFA strips and includes a holder fitted for holding and aligning strips. At the top of the dark box there is a window that aligns with a smartphone camera and a phone holder to hold the smartphone in place, both of which can be modified to accommodate different phone models. 3D-printing coordinates are available at https://www.thingiverse.com/thing:5178342/files.

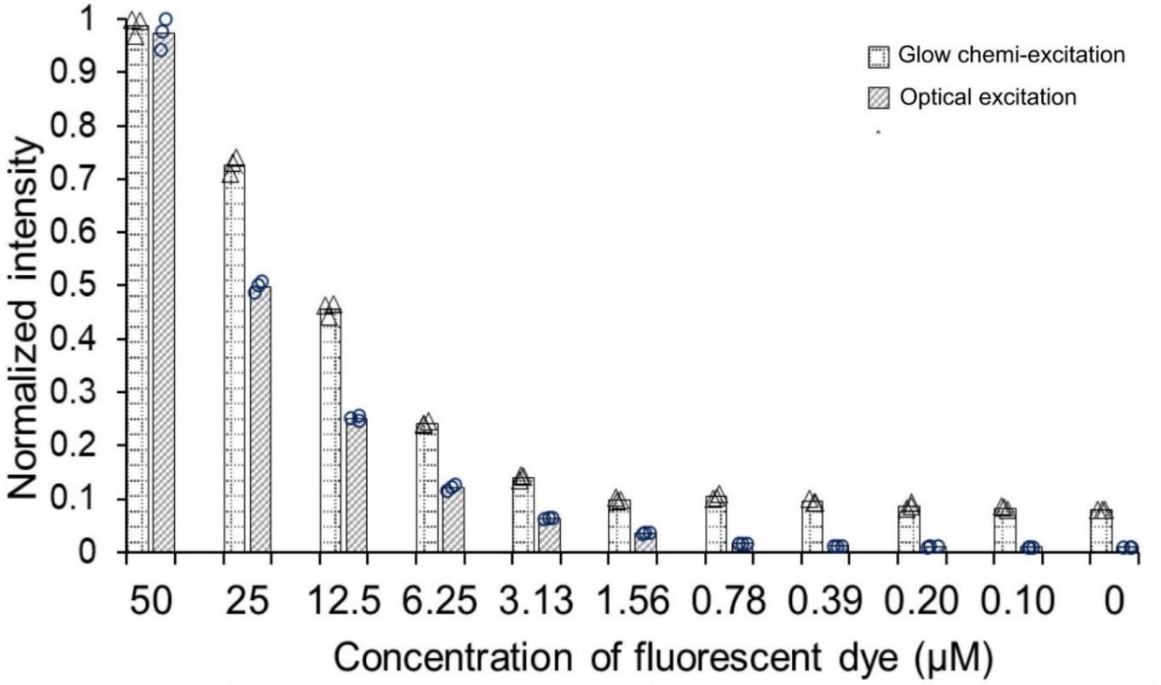

Supplementary Figure 3. Glow chemi-excitation and optical excitation of 9,10-diphenylanthracene dye. Detectability of chemi-excited fluorescent dye (9,10-diphenylanthracene) by a smartphone camera versus optically-excited fluorescent dye by a Tecan M200PRO plate reader. The bars represent the average (n=3) at each concentration and individual points are shown as triangles for glow chemi-excitation and circles for optical excitation. Fluorescent dye (stock solution made in tetrahydrofuran) was 2-fold serially diluted in butyl benzoate and 50 μL pipetted into the wells of a white (chemi-excitation) or black (optical excitation) 96-well plate. The dye was then optically excited and emitted light was read using a plate reader with Excitation 365 nm/Emission 415 nm. The dye was chemi-excited with glow excitation solution (50 μL) and imaged with a smartphone. Intensity profiles for chemi-excited particles were extracted from the smartphone images using ImageJ as described in Methods. 200 nM of dye is detectable above background (signal > blank + 3 Std Dev of blank) using glow chemi-excitation, while 390 nM is detectable above background using optical excitation.

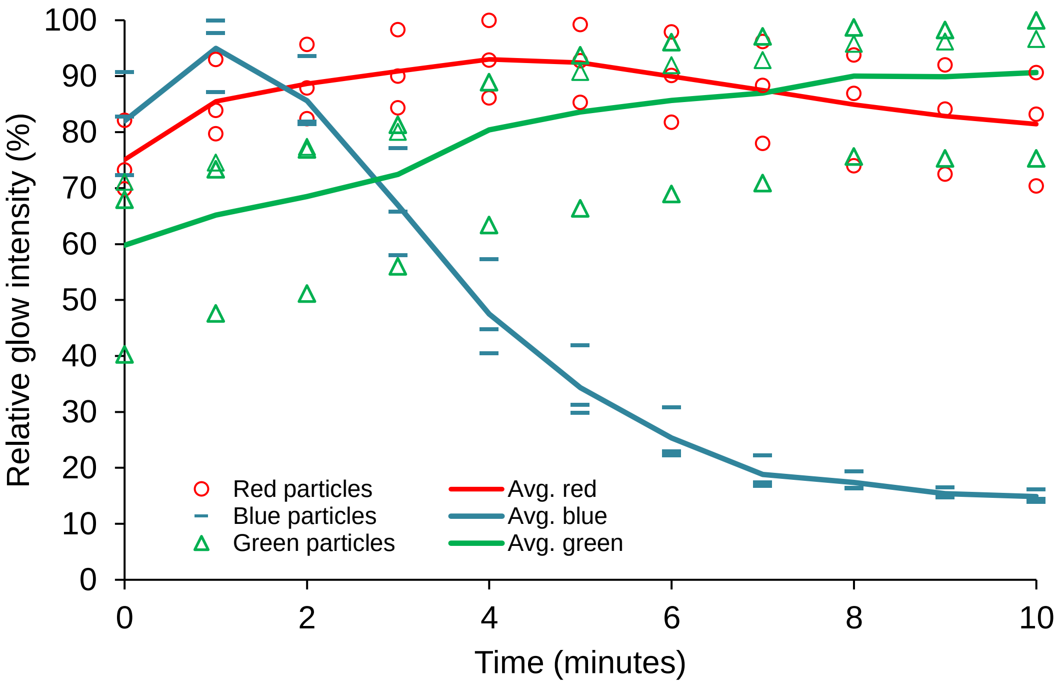

Supplementary Figure 4. Glow signal stability over time. Glow compatible fluorescent particles, red (R), green (G), and blue (B), (2× 10^9 particles) were added to the wells of a 96-well microplate (n=3; well diameter: 5 mm), chemi-excited with glow excitation solution and imaged with a Samsung Note 8 camera. Images were taken every minute for 10 minutes. Each image was analyzed using ImageJ. The intensity values for each set of replicates were normalized to the maximum value for that color and all three replicates were plotted; the line connecting the average of the three replicates was drawn to aid in visualization.

Solvent name	Toxicity	Stability of TCPO	Strength/ duration	Odor
Diethyl phthalate	Some potential	High (>1 month)	High / > 5 min	Odorless
Ethyl acetate	Low	Low (<2 weeks)	High / < 5 min	High, ethereal, fruity
Tributyl O-acetylcitrate	Low	High (>1 month)	Low / > 5 min	Slight, sweet
2-(Acetyloxy)-1-(hydroxy methyl)ethyl acetate	Low	High (>1 month)	Low /< 5 min	Low
Tributyl citrate	Low	High (>1 month)	Medium / > 5 min	Very faint herbal
Butyl benzoate	Low	High (>1 month)	High / > 5 min	Mild amber balsam
**Co-Solvent name**	**Toxicity**	**Stability of H_2O_2**	**Strength/ duration**	**Odor**
Methanol	High	High (>1 month)	High / < 5 min	Medium, alcoholic
Tert-butanol	Low	High (>1 month)	High / > 5 min	Camphorous

Supplementary Table 1. **Candidate TCPO solvents.** Solvents were screened for toxicity, stability, signal strength, and odor. The final solvent was 66.6% tributyl citrate and 33.3% butyl benzoate because they both have low toxicity and gave strong glow signals. Tert-butanol was chosen as the cosolvent for H_2O_2 because it produced a strong and longer-lasting signal.

a

	0 pg/mL	9.7 pg/mL	18 pg/mL	39 pg/mL	78 pg/mL	156 pg/mL	312 pg/mL	625 pg/mL
TL	0.00	0.00	0.00	73.90	118.85	188.14	410.92	615.51
CL	4388.65	4533.47	4212.36	4039.50	4264.38	3780.31	4154.96	4308.38
TL/CL	0.00	0.00	0.00	0.02	0.03	0.05	0.10	0.14

b

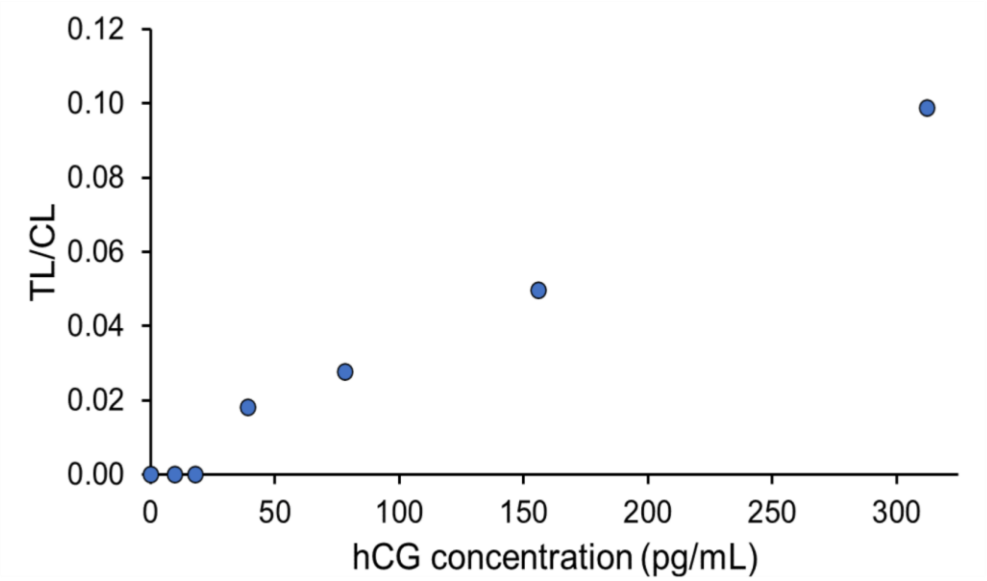

Supplementary Figure 5. Fluorescence-based LFA detection of hCG. hCG was serially diluted and detected in LFA strips using blue in-house dyed fluorescence particles. Before chemi-exciting the strips with glow excitation solution (data shown in Fig. 3), the strips were optically excited and imaged using a FluorChem system. **a** A typical image from one replicate was analyzed using ImageJ and the values for the integrated area under the curve for each peak (TL and CL) and their ratio were obtained from the intensity profiles. **b** Fluorescent LFA (integrated area ratio: TL/CL) at different hCG concentrations.

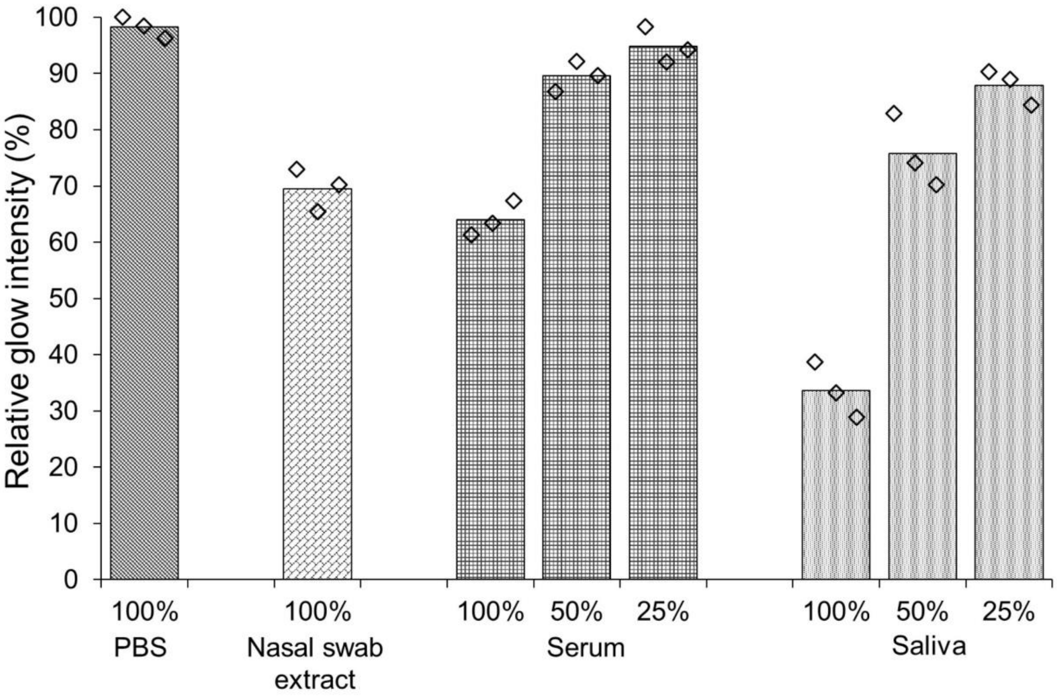

Supplementary Figure 6. Chemi-excitation of glow particles in biological matrices. 1 x 10^9 blue Fluospheres were spiked into 1x PBS (control), nasal swab extract, 100%, 50% or 25% serum (diluted in 1x PBS), and 100%, 50% or 25% saliva (diluted in 1x PBS) in wells of a 96-well white polypropylene plate (50 µL volume; n=3). Glow excitation reagent (50 µL per well) was added to each well and mixed, then imaged using a Samsung Note 8 camera as described in Methods. The pixel intensity was calculated for each well and normalized to the average value in 1x PBS. Average normalized values are shown as a bar, and individual replicates are shown as diamonds. The signal in nasal swab extract was 70% of the signal in 1x PBS, the signal in 25% saliva was 88% of the signal in 1x PBS, and the signal in 25% serum was 95% of the signal in 1x PBS, suggesting broad applicability of glow reporters.

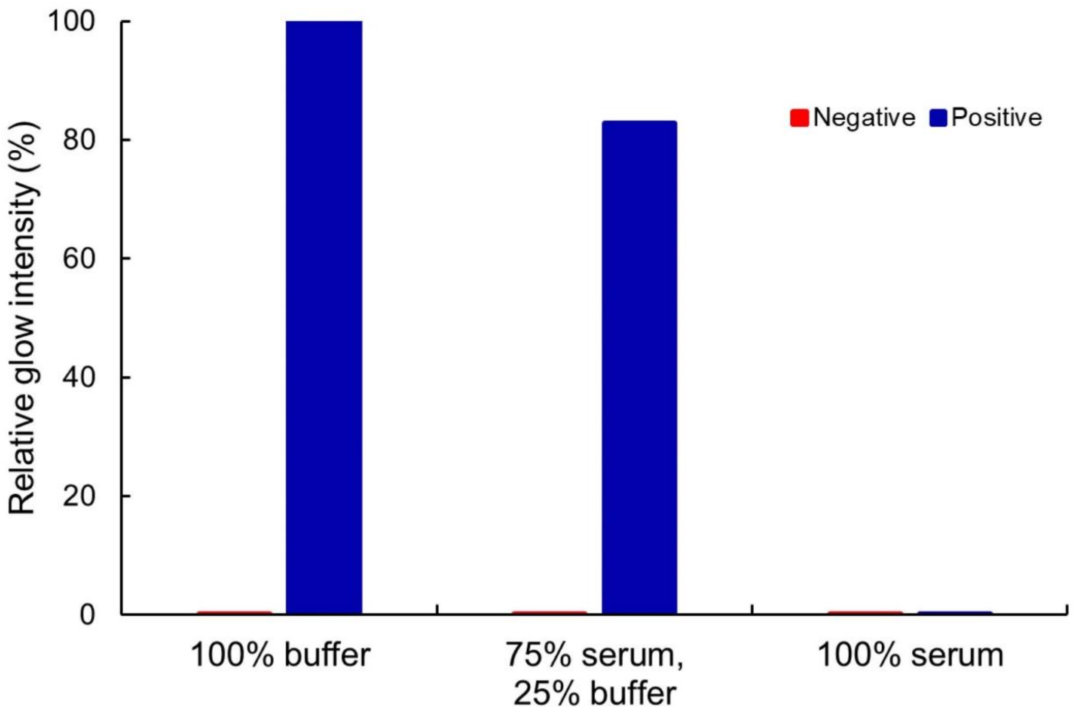

Supplementary Figure 7. Glow chemistry tolerance of 75% human serum in LFA. hCG protein (1.2 ng/mL) was spiked in human serum diluted in LFA running buffer. Samples were mixed with glow reporters, run on LFA half strips, chemi-excited with glow chemistry and imaged with a Samsung Note 8 smartphone. hCG-free negative control samples were also run in serum. Image analysis was done using in-house image analysis software. Intensity profiles along the length of the LFA strips were plotted and intensity values of the test and control lines were determined by calculating the area under the curve. Test line intensities normalized to the corresponding values in 100% buffer for negative and positive samples are shown here. Test line intensities in the negative samples were zero in all cases, no non-specific binding was observed. Glow chemistry signal in LFA is only mildly affected by the presence of 75% human serum, suggesting glow LFA compatibility with serum.

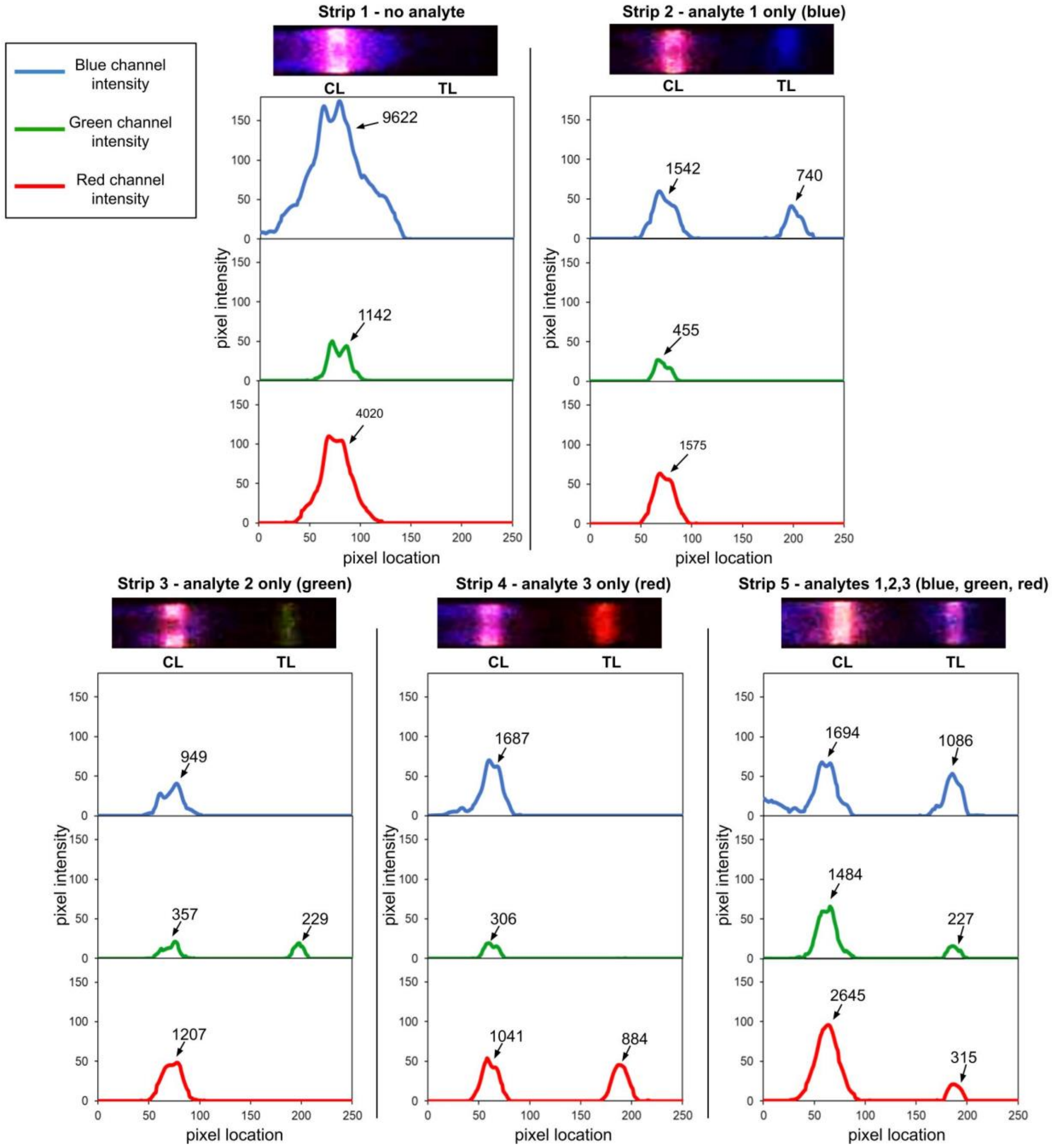

Supplementary Figure 8. Intensity analysis of glow multiplex LFA. The glow multiplex RGB image (Figure 6) was split into three channels- red, green, and blue - using ImageJ. The pixel intensities for each strip in each channel and areas under the curve for the control lines and test lines were measured using in-house software (see Methods). The pixel intensity values were copied into Microsoft Excel and plotted. As shown above, the individual analytes are only detectable (non-zero TL value) in the channel that corresponds to the detector particles' color, suggesting that multiple analytes can be detected simultaneously using color deconvolution.
