# Supplementary material for: Glowstick-inspired smartphone-readable reporters for sensitive, multiplexed lateral flow immunoassays
Source: Commun Eng. 2023 Jun 23;2:31. doi: 10.1038/s44172-023-00075-2 (PMC10955955; doi:10.1038/s44172-023-00075-2)
Supplement: Supplementary file 1 [file 44172_2023_75_MOESM2_ESM.pdf]

Description of Additional Supplementary File

File name: Supplementary Data 1

Description: Source data underlying the graphs presented in the main and supplementary figures are included in “Supplementary Data 1”.
