# Supplementary material for: Glowstick-inspired smartphone-readable reporters for sensitive, multiplexed lateral flow immunoassays
Source: Commun Eng. 2023 Jun 23;2:31. doi: 10.1038/s44172-023-00075-2 (PMC10955955; doi:10.1038/s44172-023-00075-2)
Supplement: Supplementary file 2 [file 44172_2023_75_MOESM4_ESM.pdf]

nature portfolio

Corresponding author(s): Richard C. Willson and Binh V. Vu

Last updated by author(s): Mar 28, 2023

Reporting Summary

Nature Portfolio wishes to improve the reproducibility of the work that we publish. This form provides structure for consistency and transparency in reporting. For further information on Nature Portfolio policies, see our Editorial Policies and the Editorial Policy Checklist.

Statistics

For all statistical analyses, confirm that the following items are present in the figure legend, table legend, main text, or Methods section.

n/a	Confirmed	
☐	☒	The exact sample size (*n*) for each experimental group/condition, given as a discrete number and unit of measurement
☒	☐	A statement on whether measurements were taken from distinct samples or whether the same sample was measured repeatedly
☒	☐	The statistical test(s) used AND whether they are one- or two-sided<br>*Only common tests should be described solely by name; describe more complex techniques in the Methods section.*
☒	☐	A description of all covariates tested
☒	☐	A description of any assumptions or corrections, such as tests of normality and adjustment for multiple comparisons
☒	☐	A full description of the statistical parameters including central tendency (e.g. means) or other basic estimates (e.g. regression coefficient) AND variation (e.g. standard deviation) or associated estimates of uncertainty (e.g. confidence intervals)
☒	☐	For null hypothesis testing, the test statistic (e.g. *F*, *t*, *r*) with confidence intervals, effect sizes, degrees of freedom and *P* value noted<br>*Give P values as exact values whenever suitable.*
☒	☐	For Bayesian analysis, information on the choice of priors and Markov chain Monte Carlo settings
☒	☐	For hierarchical and complex designs, identification of the appropriate level for tests and full reporting of outcomes
☒	☐	Estimates of effect sizes (e.g. Cohen's *d*, Pearson's *r*), indicating how they were calculated

Our web collection on statistics for biologists contains articles on many of the points above.

Software and code

Policy information about availability of computer code

Data collection	Fluorescent particles were measured with the Tecan M200PRO microplate reader using the instrument software.<br><br>Fluorescent LFA strips were imaged with a Samsung Note 8 smartphone camera. Images were taken using the built-in Samsung camera Pro mode with manually set exposure time (2-10 sec), white balance set to 5500 K, ISO set to 800 (maximum), and manually focused. Fluorescent LFA strips were also imaged on a gel documentation system composed of a FluorChem SP gel cabinet (Alpha Innotech Corp., San Leandro CA) and a CoolSNAP K4 CCD 2,048 × 2,048-pixel camera (Photometrics, Blaine WA) controlled by Micro-Manager 1.4.22 software (Vale Lab, University of California, San Francisco).<br><br>Gold LFA strips were photographed with an iPhone XR or scanned on an Epson Perfection V600 scanner.

Data analysis	The Tecan reader software automatically exports the data in an Excel spreadsheet. The data was further processed and plotted using Microsoft Excel.<br><br>Intensity profiles were extracted from the smartphone, the FluorChem gel documentation system or the scanner images using NIH ImageJ or the in-house developed LFA Image Analysis software. The data was further processed and plotted using Microsoft Excel software.<br><br>The in-house LFA Image Analysis software is freely available from our GitHub account: https://github.com/willsonlab/LFA-Image-Analysis

For manuscripts utilizing custom algorithms or software that are central to the research but not yet described in published literature, software must be made available to editors and reviewers. We strongly encourage code deposition in a community repository (e.g. GitHub). See the Nature Portfolio guidelines for submitting code & software for further information.

Data

Policy information about availability of data

All manuscripts must include a data availability statement. This statement should provide the following information, where applicable:

- Accession codes, unique identifiers, or web links for publicly available datasets
- A description of any restrictions on data availability
- For clinical datasets or third party data, please ensure that the statement adheres to our policy

The data supporting the findings of this work are available within the article and its Supplementary Information. The source data generated in this study are available in the Supplementary Data.

Human research participants

Policy information about studies involving human research participants and Sex and Gender in Research.

Reporting on sex and gender	Not necessary for this study.<br>The PI and 2 senior members of his research group volunteered to donate samples for spiking studies during LFA development.
Population characteristics	Not necessary for this study.<br>The PI and 2 senior members of his research group volunteered to donate samples for matrix interference and spiking studies during LFA development.
Recruitment	No recruitment was done. Specimens were self-collected by a PI and 2 senior members of his research group.
Ethics oversight	University of Houston IRB committee

Note that full information on the approval of the study protocol must also be provided in the manuscript.

Field-specific reporting

Please select the one below that is the best fit for your research. If you are not sure, read the appropriate sections before making your selection.

☒ Life sciences ☐ Behavioural & social sciences ☐ Ecological, evolutionary & environmental sciences

For a reference copy of the document with all sections, see nature.com/documents/nr-reporting-summary-flat.pdf

Life sciences study design

All studies must disclose on these points even when the disclosure is negative.

Sample size	Collected nasal extracts and saliva samples were pooled. No statistical method was used to determine sample size; it was not necessary for this study.
Data exclusions	Specimens were self-collected by adult lab members asymptomatic and negative by EUA'd LFA for COVID-19. No data were excluded.
Replication	At least 3 technical replicates (to assess the variation of the assay and/or sample preparation) were performed for each sample. We have reproduced the results reported in the manuscript multiple times.
Randomization	Randomization was not done in this study.
Blinding	Blinding was not done in this study.

Reporting for specific materials, systems and methods

We require information from authors about some types of materials, experimental systems and methods used in many studies. Here, indicate whether each material, system or method listed is relevant to your study. If you are not sure if a list item applies to your research, read the appropriate section before selecting a response.

Materials & experimental systems

n/a	Involved in the study
☐	☒ Antibodies
☒	☐ Eukaryotic cell lines
☒	☐ Palaeontology and archaeology
☒	☐ Animals and other organisms
☒	☐ Clinical data
☒	☐ Dual use research of concern

Methods

n/a	Involved in the study
☒	☐ ChIP-seq
☒	☐ Flow cytometry
☒	☐ MRI-based neuroimaging

Antibodies

Antibodies used

mouse anti-βhCG antibodies (Arista Biologics; clone 2; #CGBCG-0402)
goat polyclonal anti-αhCG antibodies (Arista; #ABACG-0500)

mouse anti-SARS-CoV-2 nucleoprotein monoclonal antibodies (Sino Biological; #40143-MM08)
rabbit anti-SARS-CoV-2 nucleoprotein (ExonBio; #NP11H9)
mouse anti-SARS-CoV-2 nucleoprotein antibodies (Bioss; #bsm-41411M)

mouse anti-prostate specific antigen antibodies (Abcam; #ab403)
goat polyclonal anti-prostate specific antigen antibodies (R&D systems; AF1344)

goat polyclonal anti-mouse antibodies (Arista; #ABGAM-0500)
goat polyclonal anti-rabbit antibodies (Arista; #ABGAR-0500)

Validation

We purchase our antibodies from reputable commercial vendors that supply detailed COAs. Our studies presented in this manuscript do not require a further validation of the antibodies used. Nevertheless, upon receipt and then on a regular basis we confirm the performance and specificity of our antibodies by running a negative and positive (at a specific analyte concentration) lateral flow test.
